# Supplementary material for: Gene Regulation Network Analysis on Human Prostate Orthografts Highlights a Potential Role for the JMJD6 Regulon in Clinical Prostate Cancer
Source: Cancers (Basel). 2021 Apr 26;13(9):2094. doi: 10.3390/cancers13092094 (PMC8123677; doi:10.3390/cancers13092094)
Supplement: Supplementary file 1 [file cancers-13-02094-s001.zip › cancers-1148422-Supplementary.pdf]

cancers

MDPI

Supplementary Material: Gene Regulation Network Analysis on Human Prostate Orthografts Highlights A Potential Role for the *JMJD6* Regulon in Clinical Prostate Cancer

Supplementary methods

Orthograft model and transcriptomic analysis

Human PCa cell lines were authenticated using the Promega GenePrint 10 System. CWR - RRID:CVCL_LI38, 22RV1 - RRID:CVCL_1045, LNCAP-RRID:CVCL_4783, LNCAPAI -RRID:CVCL_4791 and VCAP - RRID:CVCL_2235. Hormone naïve cell lines CWR LNCAP and VCAP were maintained in RPMI 1640 with 2 mmol Glutamine and 10% Foetal Bovine Serum (FBS). Charcoal Stripped FBS (CSS) was used for maintenance of CR cell lines 22RV1 and LNCAPAI. 14×106 PC cells in serum free RPMI were mixed with matrigel (1:1), with final volume of 50 µl, and orthotopically injected into the anterior prostate of 10 week old male CD-1 Nude mice (Charles River Labs) +/- surgical castration (Project Licence P5EE22AEE), reviewed by local ethics committee in full compliance with UK Home Office regulations (UK Animals (Scientific Procedures) Act 1986).

In vivo experiments were performed in accordance with the ARRIVE guidelines, and were reviewed by a local ethics committee under the Project Licence P5EE22AEE in full compliance with the UK Home Office regulations (UK Animals (Scientific Procedures) Act 1986). Prostate cancer cells were suspended in serum-free RPMI medium and mixed 1:1 with Matrigel (Corning, NY, USA). Briefly, 14×106 cells (in 50 µl) were injected into the anterior prostate of CD1-nude mice (Charles River Laboratories, Wilmington, MA, USA). For CRPC conditions, orchidectomy was performed at the time of injection. Tumour growth was monitored weekly using A Vevo3100 ultrasound imaging system (Fujifilm Visualsonics, The Netherlands). Tumours were then allowed to grow for 9 weeks before reaching endpoint. At the end of the experiment, tumour orthografts were collected and weighted. Half of the tumour material was fixed in 10% formalin for histological procedures and the other half was snap-frozen in liquid nitrogen for transcriptomic analysis.

Frozen tumours were manually crushed, reduced into powder and further processed using QIAshredder homogeniser columns (Qiagen, Hilden, Germany) before extraction. For cells, RNA was extracted 72 hours after initial seeding, when cells reached around 80% confluence. RNA extraction was performed using RNeasy Mini Kit (Qiagen, Hilden, Germany) with on-column DNase digestion (RNase-Free DNase Set, Qiagen, Hilden, Germany). Quality of the purified RNA was tested on an Agilent 2200 Tapestation using RNA screentape.

Fastq pre-processing

UGLA [1], UTA [2] and EMC [3] fastq files underwent the following steps: adapter trimming using 'ILLUMINACLIP' step from 'Trimmomatic' [4] version 0.36; alignment to GRCh37.75 human reference (and GRCm38.p4 mouse reference for orthografts) with TopHat [5]v2.0.14; gene level raw counts calculation using 'HTSeq' [6] version 0.9.1 and GRCh37.75 (and GRCm38.p4 for orthografts) annotation files (.gtf) for human and mouse reads respectively; gene and transcripts level FPKM quantification using cufflinks-2.2.1 [7] and Ensembl's GRCh37.75 (and GRCm38.p4 for orthografts) gtf files for human and mouse reads respectively.

ICGC RNA-seq reads were previously processed [8] through alignment to hg1000 using BWA (v.0.5.9-r16 for reads up to 51 bases and v. 0.7.7-r441 for reads with 100 bases) and SAMtools. Uniquely mapped reads were annotated using Ensembl v62. Gene expression levels were quantified in reads per kilobase of exon per million mapped reads

(RPKM). For prognostic analysis for the *JMJD6* regulon in the ICGC cohort, enrichment of regulons was performed using pre-processed gene level raw read counts.

Quality control of sequencing data

Both unaligned and mapped reads from UGLA, UTA and EMC samples (for which raw fastq files were available) underwent quality control. FastQC v0.11.9 has been used to check sequence quality, base content, GC content, N content and duplication levels. Rseqc v2.6.4 has been adopted to check mapped gene body coverage, inner distance, and read duplications. All the investigated samples passed the quality checks aside from 'PC_9324' from UTA cohort given the skewed mapped reads coverage towards the end of the genes, resulting 27 informative tumour samples in the UTA cohort.

Counts matrices

Matrices of normalised counts were obtained by pooling FPKM profiles from the UGLA set (orthografts). Gene level raw counts from the clinical datasets (UTA, EMC, ICGC) were processed with Deseq2 [9] v1.26 normalisation, through the 'DESeqDataSet-FromMatrix' and then 'DESeq' functions, by applying default parameters.

Differential expression analysis

For each of the clinical cohorts (namely UTA, EMC and ICGC respectively), the fold changes of each differentially expressed gene (DEG) were calculated on a per-sample basis from the ratio of Deseq2 normalised counts for the tumoral sample and the average Deseq2 normalised counts of the control benign prostatic samples within the dataset, and is expressed in a log2 scale. The significance of the ratio was calculated through the interpolation on the standardised Gaussian distribution, after dividing each counts difference (Deseq2 norm counts of tumour minus Deseq2 norm counts of the normal group) by the standard deviation of the Deseq2 norm counts in the panel of normal samples. Deseq2 normalised counts were extracted with the Deseq2 function 'counts()' by setting the 'normalized' parameter to 'True'. P-values were adjusted using the function 'p.adjust()' from the R stat package v4.0.3, by setting the 'method' parameter to 'fdr'. For the analysis of DEGs for each sample, a threshold of FDR was set at adjusted p-value ≤0.05 and $\log_2$ fold change above or below zero have been used to identify genes up or down-regulated, respectively.

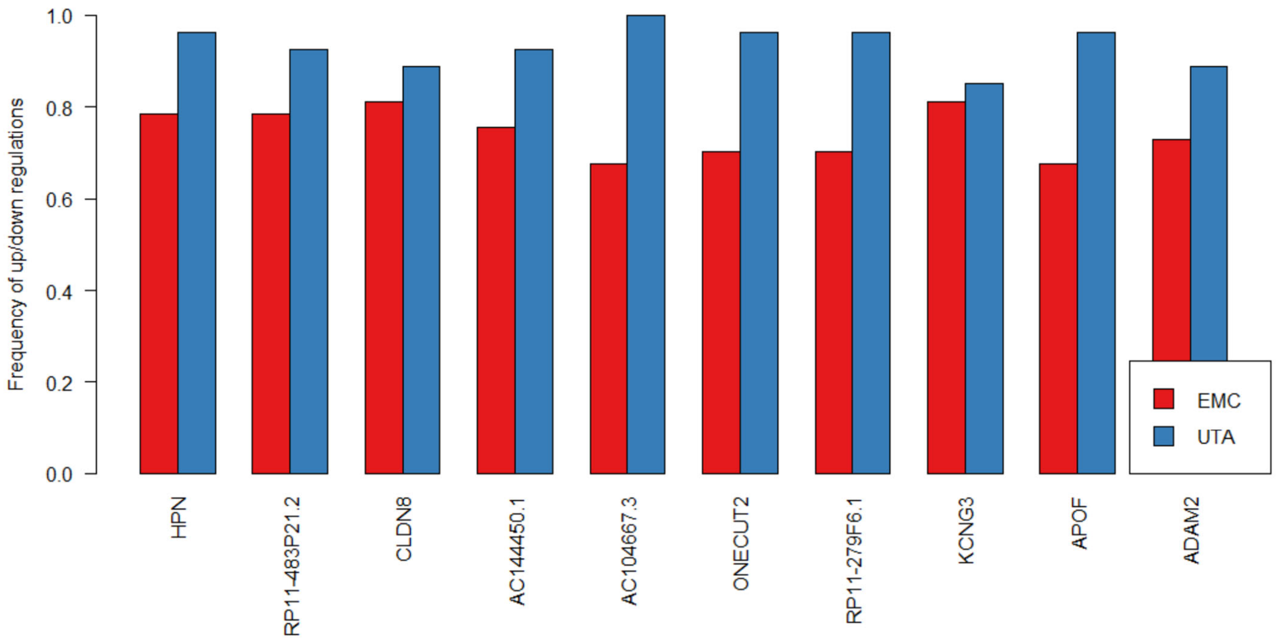

(**a**)

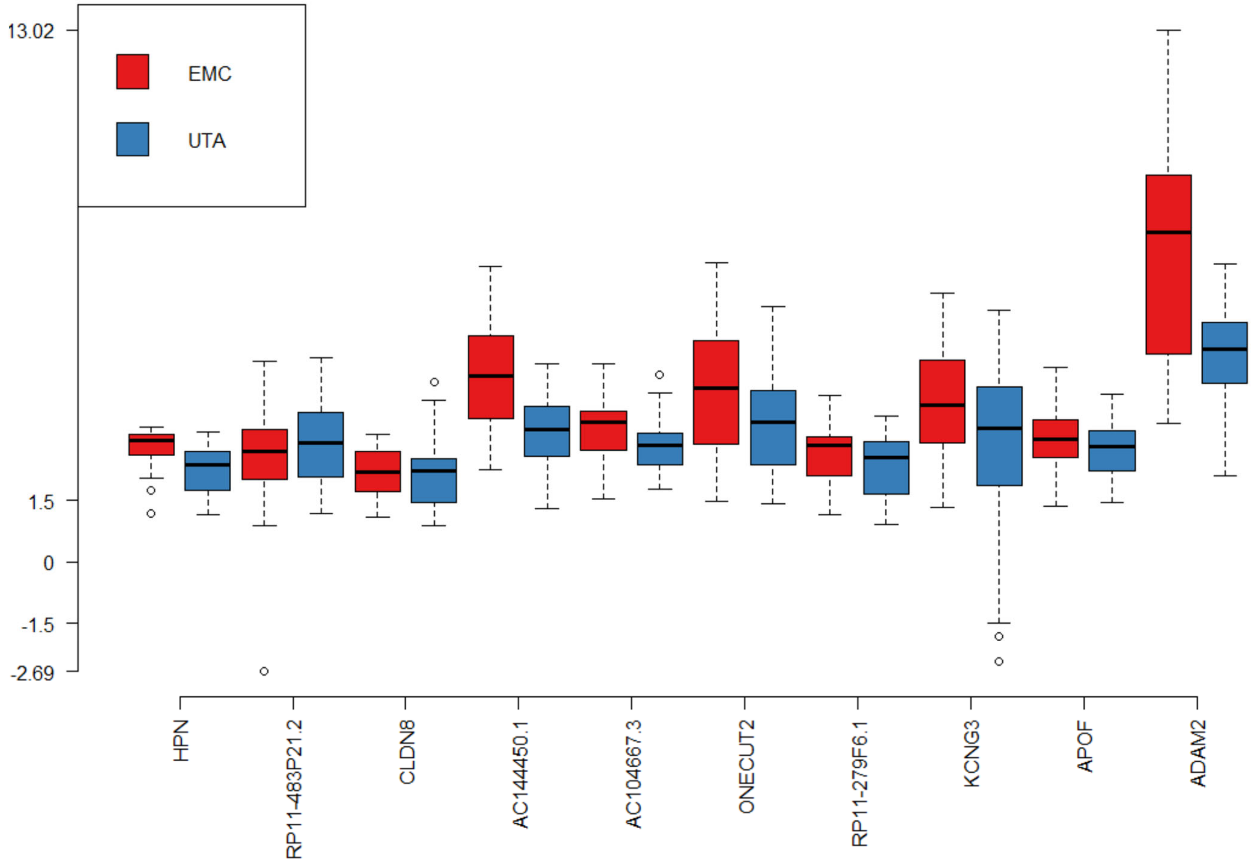

(**b**)

Figure S1. Top ten differentially expressed genes (DEG) in the UTA and EMC clinical cohorts. **(A)** Frequency of coherent alterations (Y-axis) is represented as the proportion of samples (in percentage) within each cohort with altered expression

of genes listed, when compared to benign control samples. (UTA in blue; EMC in red). Differentially expressed genes were calculated on a per-sample basis for each clinical cohort. The relative frequency of samples in which the gene was found downregulated was subtracted from the relative frequency of samples in which the gene was found upregulated. (**B**) Frequency of coherent alterations (Y axis) is represented as log2 fold change distributions in the clinical tumours for the two cohorts.

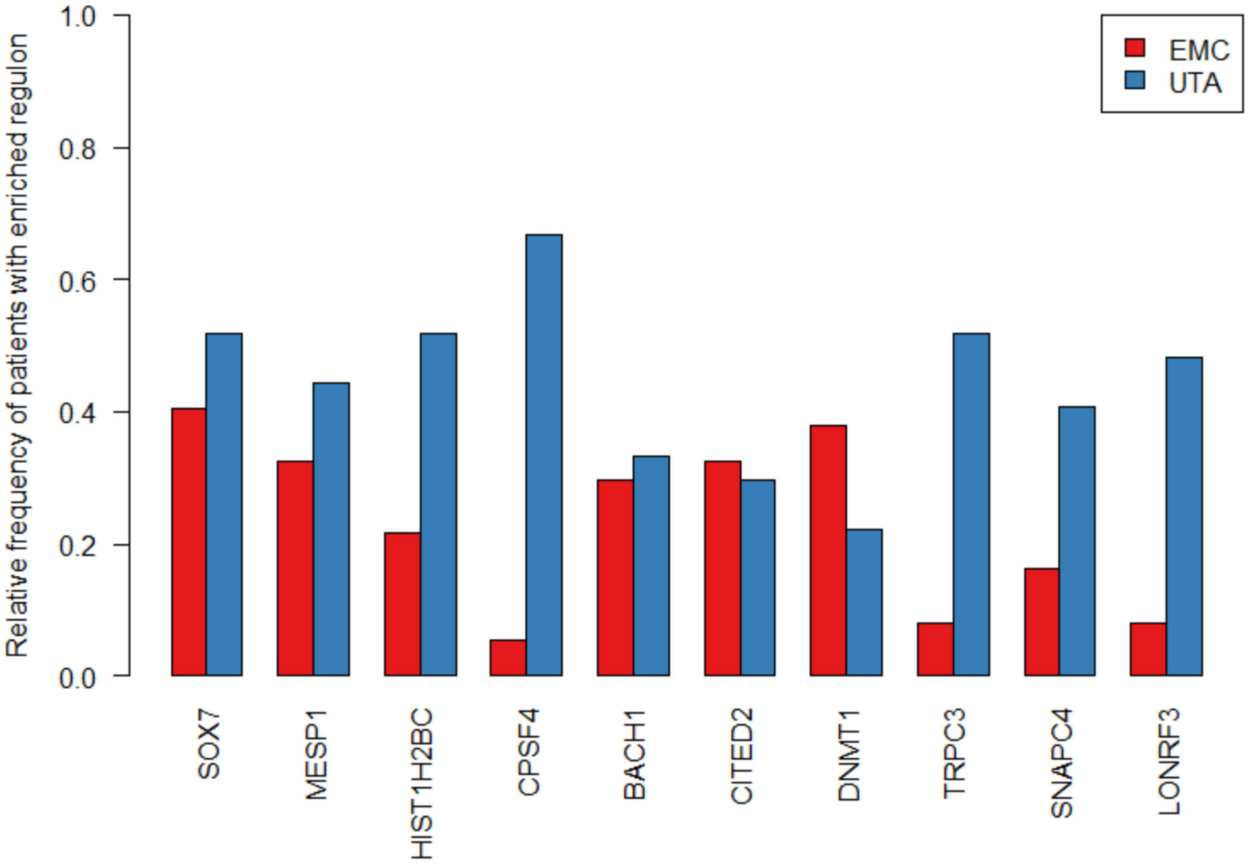

Figure S2. Top 10 active regulons in the UTA and EMC clinical cohorts. The Y-axis shows the frequency of enrichment of the regulons within the clinical cohorts. Active regulons were ranked by average frequency of enrichment.

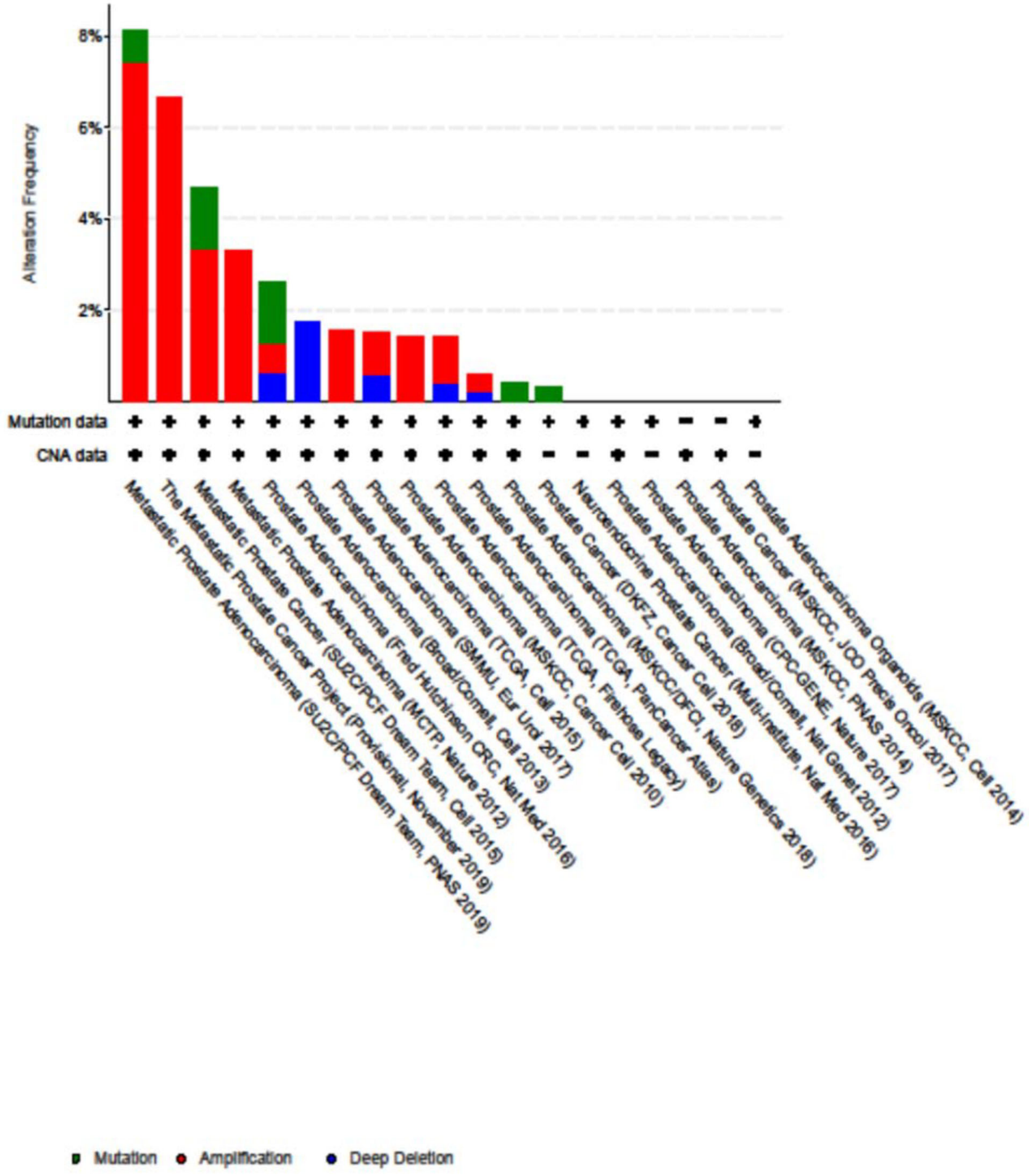

Figure S3. Alteration frequency (Y-axis) for *JMJD6* gene across all 22 prostate cancer datasets in the c-Bio portal. (Colour – green, mutation; red, amplification, blue, deep deletion.).

References

1. M.J. Salji, Quantitative Proteomics and Metabolomics of Castration Resistant Prostate Cancer, University of Glasgow, 2018.
2. A. Ylipää, K. Kivinummi, A. Kohvakka, M. Annala, L. Latonen, M. Scaravilli, et al., Transcriptome sequencing reveals PCAT5 as a Novel ERG-Regulated long Noncoding RNA in prostate cancer, Cancer Res. 75 (2015) 4026–4031.
3. S. Chen, V. Huang, X. Xu, J. Livingstone, F. Soares, J. Jeon, et al., Widespread and Functional RNA Circularization in Localized Prostate Cancer, Cell. 176 (2019) 831-843.e22.

4. A.M. Bolger, M. Lohse, B. Usadel, Trimmomatic: a flexible trimmer for Illumina sequence data., Bioinformatics. 30 (2014) 2114–2120.
5. C. Trapnell, L. Pachter, S.L. Salzberg, TopHat: Discovering splice junctions with RNA-Seq, Bioinformatics. 25 (2009) 1105–1111.
6. S. Anders, P.T. Pyl, W. Huber, HTSeq--a Python framework to work with high-throughput sequencing data., Bioinformatics. 31 (2015) 166–169.
7. C. Trapnell, A. Roberts, L. Goff, G. Pertea, D. Kim, D.R. Kelley, et al., Differential gene and transcript expression analysis of RNA-seq experiments with TopHat and Cufflinks., Nat. Protoc. 7 (2012) 562–578.
8. C. Gerhauser, F. Favero, T. Risch, R. Simon, L. Feuerbach, Y. Assenov, et al., Molecular Evolution of Early-Onset Prostate Cancer Identifies Molecular Risk Markers and Clinical Trajectories, Cancer Cell. 34 (2018) 996-1011.e8.
9. M.I. Love, W. Huber, S. Anders, Moderated estimation of fold change and dispersion for RNA-seq data with DESeq2, Genome Biol. 15 (2014) 1–21.
